# Salvaging low contrast abdominal CT studies using noise-optimised virtual monoenergetic image reconstruction

**DOI:** 10.1259/bjro.20220006

**Published:** 2022-05-10

**Authors:** Scherwin Mahmoudi, Marvin Lange, Lukas Lenga, Ibrahim Yel, Vitali Koch, Christian Booz, Simon Martin, Simon Bernatz, Thomas Vogl, Moritz Albrecht, Jan-Erik Scholtz

**Affiliations:** ^1^ Department of Diagnostic and Interventional Radiology, University Hospital Frankfurt, Theodor-Stern-Kai, Frankfurt, Germany

## Abstract

**Objectives:**

To assess the impact of noise-optimised virtual monoenergetic imaging (VMI+) on image quality and diagnostic evaluation in abdominal dual-energy CT scans with impaired portal-venous contrast.

**Methods:**

We screened 11,746 patients who underwent portal-venous abdominal dual-energy CT for cancer staging between 08/2014 and 11/2019 and identified those with poor portal-venous contrast.

Standard linearly-blended image series and VMI+ image series at 40, 50, and 60 keV were reconstructed. Signal-to-noise ratio (SNR) and contrast-to-noise ratio (CNR) of abdominal organs and vascular structures were calculated. Image noise, image contrast and overall image quality were rated by three radiologists using 5-point Likert scale.

**Results:**

452 of 11,746 (4%) exams were poorly opacified. We excluded 190 cases due to incomplete datasets or multiple exams of the same patient with a final study group of 262. Highest CNR values in all abdominal organs (liver, 6.4 ± 3.0; kidney, 17.4 ± 7.5; spleen, 8.0 ± 3.5) and vascular structures (aorta, 16.0 ± 7.3; intrahepatic vein, 11.3 ± 4.7; portal vein, 15.5 ± 6.7) were measured at 40 keV VMI+ with significantly superior values compared to all other series. In subjective analysis, highest image contrast was seen at 40 keV VMI+ (4.8 ± 0.4), whereas overall image quality peaked at 50 keV VMI+ (4.2 ± 0.5) with significantly superior results compared to all other series (*p* < 0.001).

**Conclusions:**

Image reconstruction using VMI+ algorithm at 50 keV significantly improves image contrast and image quality of originally poorly opacified abdominal CT scans and reduces the number of non-diagnostic scans.

**Advances in knowledge:**

We validated the impact of VMI+ reconstructions in poorly attenuated DECT studies of the abdomen in a big data cohort.

## Introduction

Sufficient contrast is essential to facilitate precise and reliable diagnosis in contrast-enhanced CT.^1,2^ Even when optimal scanning conditions are provided, suboptimal contrast is frequently encountered in CT. This is regularly seen in obese patients with high distribution area of iodine or in patients with low cardiac output, in whom reduced iodine flux may decrease contrast in CT scans.^3–5^ Those cases require pre- and post-exam optimisation to provide sufficient contrast for optimal image evaluation.^6,7^ In addition, medical conditions that require a reduction of contrast media such as renal impairment or allergy to iodine contrast media are commonly encountered in clinical practice and are associated with insufficient contrast.^8–10^

Several studies have demonstrated that contrast-enhanced scans increase diagnostic accuracy in the detection of malignant abdominal lesions.^11,12^ Where poor contrast opacification in CT scans limits an accurate detection of lesions, a retrospective approach to enhance contrast conditions in non-diagnostic scans may be helpful.

Dual-energy computed tomography (DECT) allows post-processing techniques that have shown to increase image contrast. Noise-optimised virtual monoenergetic imaging (VMI+) is a recently developed post-processing technique to optimise image contrast and has been shown to improve image quality compared to standard linearly-blended imaging or traditional virtual monoenergetic imaging (VMI) algorithms. VMI+ allows for a separation of reconstruction data in low and optimal kiloelectronvolt (keV) settings and a further splitting of these data sets into low and high frequency data sets. By combining the lower spatial frequency data set obtained at low energy levels and the high spatial frequency data set obtained at optimal energy levels, increased iodine signal attenuation can be achieved while maintaining moderately low noise levels.^13–15^ One of the key advantages of the VMI+ algorithm is that it can be applied retrospectively, *e.g.* in cases with impaired contrast.^16^

Several small studies have already demonstrated improved image quality using VMI+ algorithms at low energy levels compared with standard linearly blended reconstructions in oncologic radiology^17–20^ as well as in emergency medicine.^21,22^ Further, VMI+ algorithm has been shown to improve visualisation of vascular anatomy and pathologies, such as pulmonary embolism, endoleaks, and aortic dissection.^23–25^

While superior image quality of VMI+ has been reported in comparison with standard linearly-blended image reconstruction in several smaller studies,^17–19^ the impact of VMI+ has not been evaluated in a large cohort yet. In this study, we validated the impact of VMI+ reconstructions on contrast conditions in poorly attenuated DECT studies of the abdomen in a large cohort.

## Methods and materials

### Patient population

This retrospective study was approved by the local institutional review board with a waiver for written consent.

**Figure 1.**
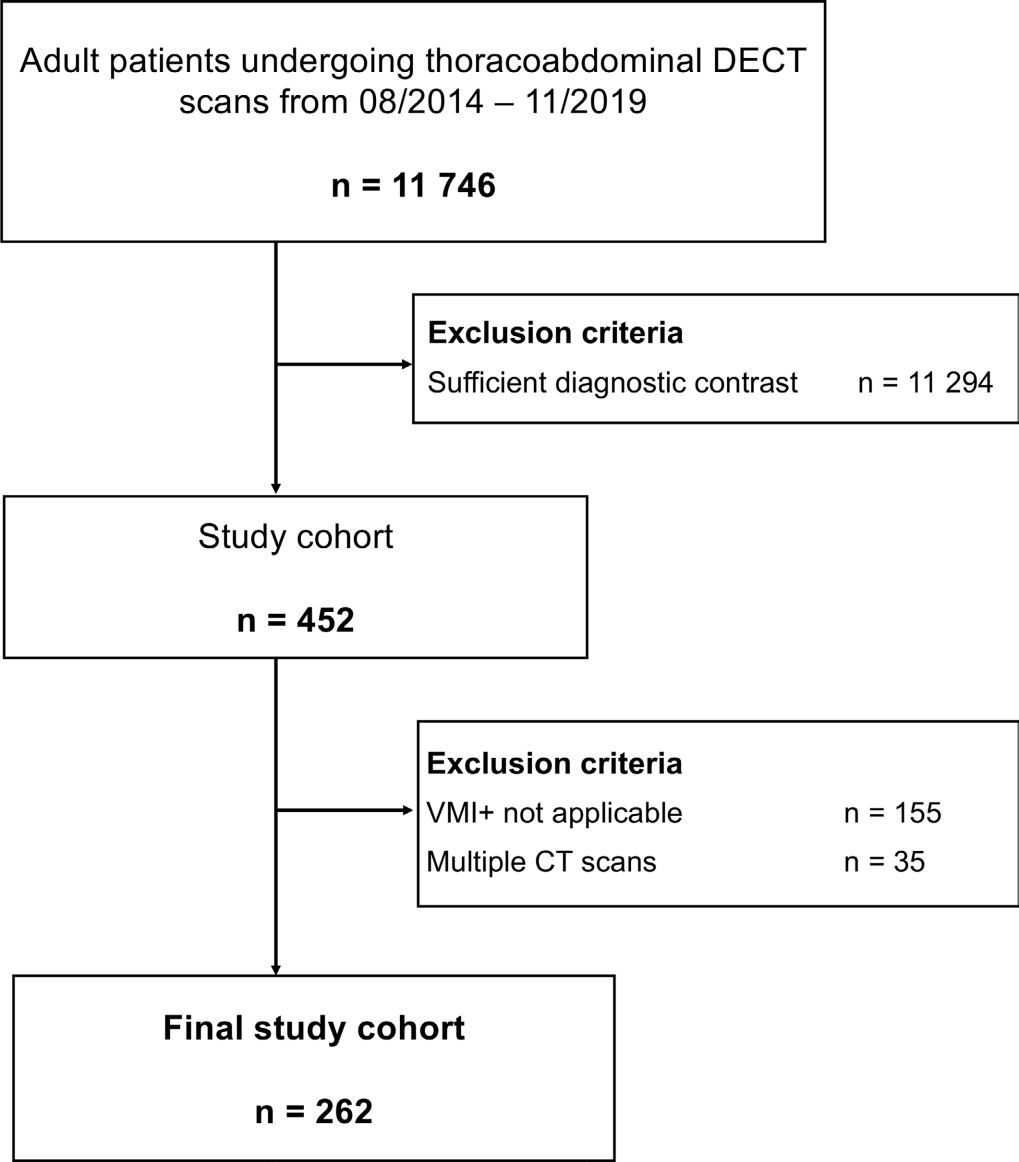
Study flow chart

We screened all patients who underwent DECT of the abdomen in portal-venous phase for cancer staging purposes between 08/2014 and 11/2019 (*n* = 11,746) and identified all cases with poor contrast based on quantitative parameters. We defined poor contrast when attenuation difference between intrahepatic veins (IHV) and liver parenchyma (LP) was less than 20 HU based on the results of prior studies which showed that malignant lesions occasionally only show subtle enhancement of 10 and 20 Hounsfield Units (HU) between unenhanced and enhanced images.^26,27^ Insufficient image contrast was identified in 452 studies (4%). In cases of patients with multiple CT studies with poor contrast, only the first CT scan with poor contrast was included. Further exclusion criteria were scans with incomplete dual-energy raw datasets consisting of low kV and high kV image stacks in thin slice thickness (1 mm). A flow chart is provided in Figure 1.

### DECT image acquisition

All exams were performed on a third-generation dual-source CT scanner (SOMATOM Force, Siemens Healthineers, Forchheim, Germany) in dual-energy mode during clinical routine.

Preset scanner settings for abdominal portal-venous CT exams were as followed: Tube A (tube voltage of 90 kVp, tube current of 95 mAs), Tube B (tube voltage of 150 kVp, tube current of 59 mAs). Rotation time was 0.5 s. An additional tin filter (Selective Photon Shield II, Siemens Healthineers) was used in Tube B to reduce radiation exposure. Image data was acquired in craniocaudal direction with a pitch of 1.0 and collimation of 2 × 192 × 0.6 mm. Automated tube current modulation (CAREdose 4D, Siemens Healthineers) was activated to adapt tube current to the patient’s habitus in real-time throughout the examination.

Images were reconstructed using iterative reconstruction algorithm (ADMIRE^®^, Siemens Healthineers, Strength Level 3).

A total of 1.2 ml/kg body weight with a maximum of 120 ml of non-ionic contrast agent (Imeron^®^ 350 mg iodine/ml; Bracco, Milan, Italy) was administered. Contrast media injection was performed through an i.v. cannula of the antecubital fossa or the forearm at a minimum flow of 2 ml s^−1^, followed by a 30-ml saline flush. Image acquisition during venous phase of contrast enhancement started 70 s after contrast agent injection in inspiratory breath-hold.

CT dose index (CTDI) and dose-length-product (DLP) were recorded from the patient protocol.

### DECT image reconstruction

During clinical routine, linear blended images were automatically reconstructed merging 60% of the low-kV spectrum with 40% of the high-kV image spectrum (M_0.6) to simulate standard single-energy 120-kv acquisition.

**Figure 2.**
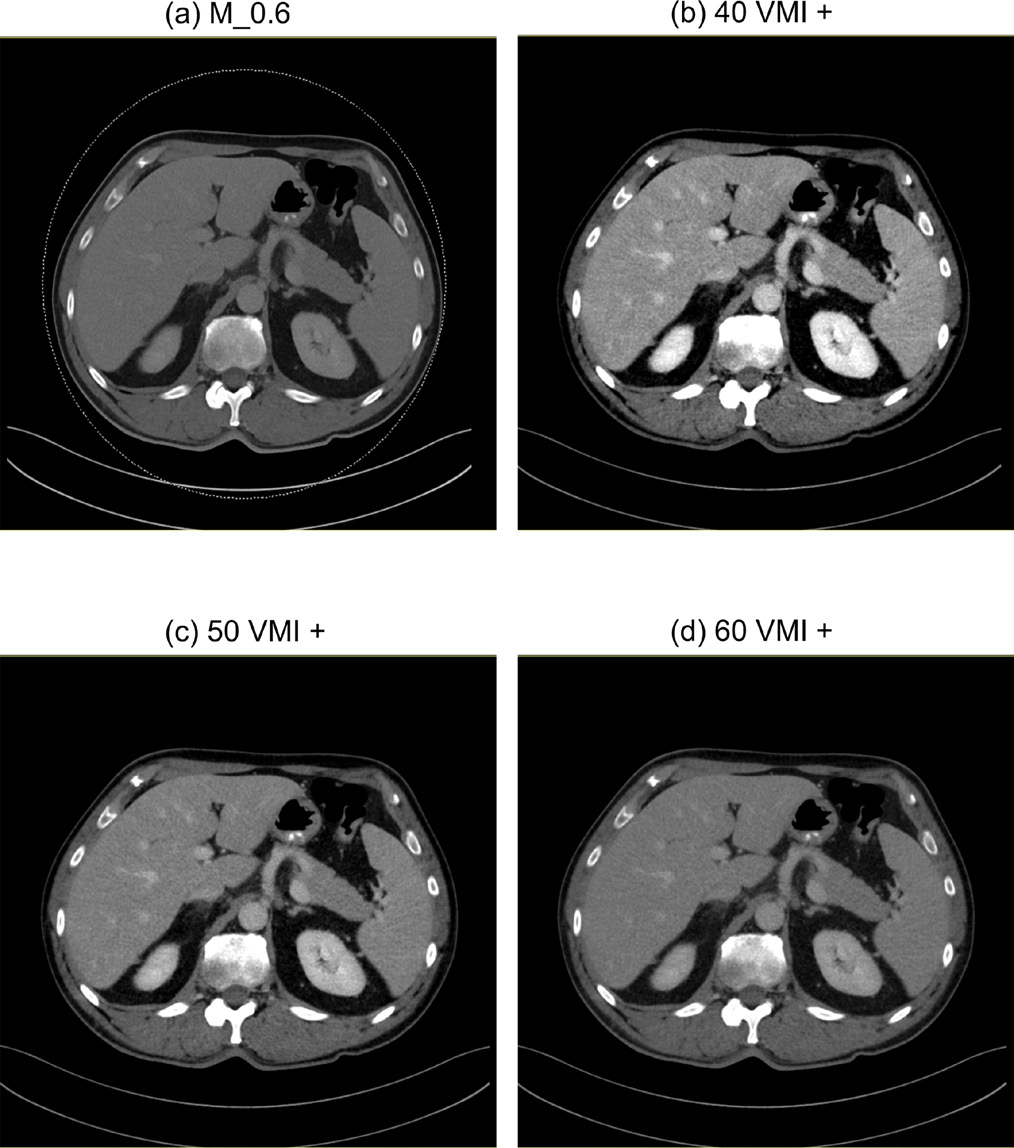
Axial DE-CT images of a 45-year-old male patient. M_0.6 (**a**), 40 VMI+ (**b**), 50 VMI+ (**c**), 60 VMI+ (**d**). Window settings in all reconstructions: level, 56 HU; width 342 HU.

**Figure 3.**
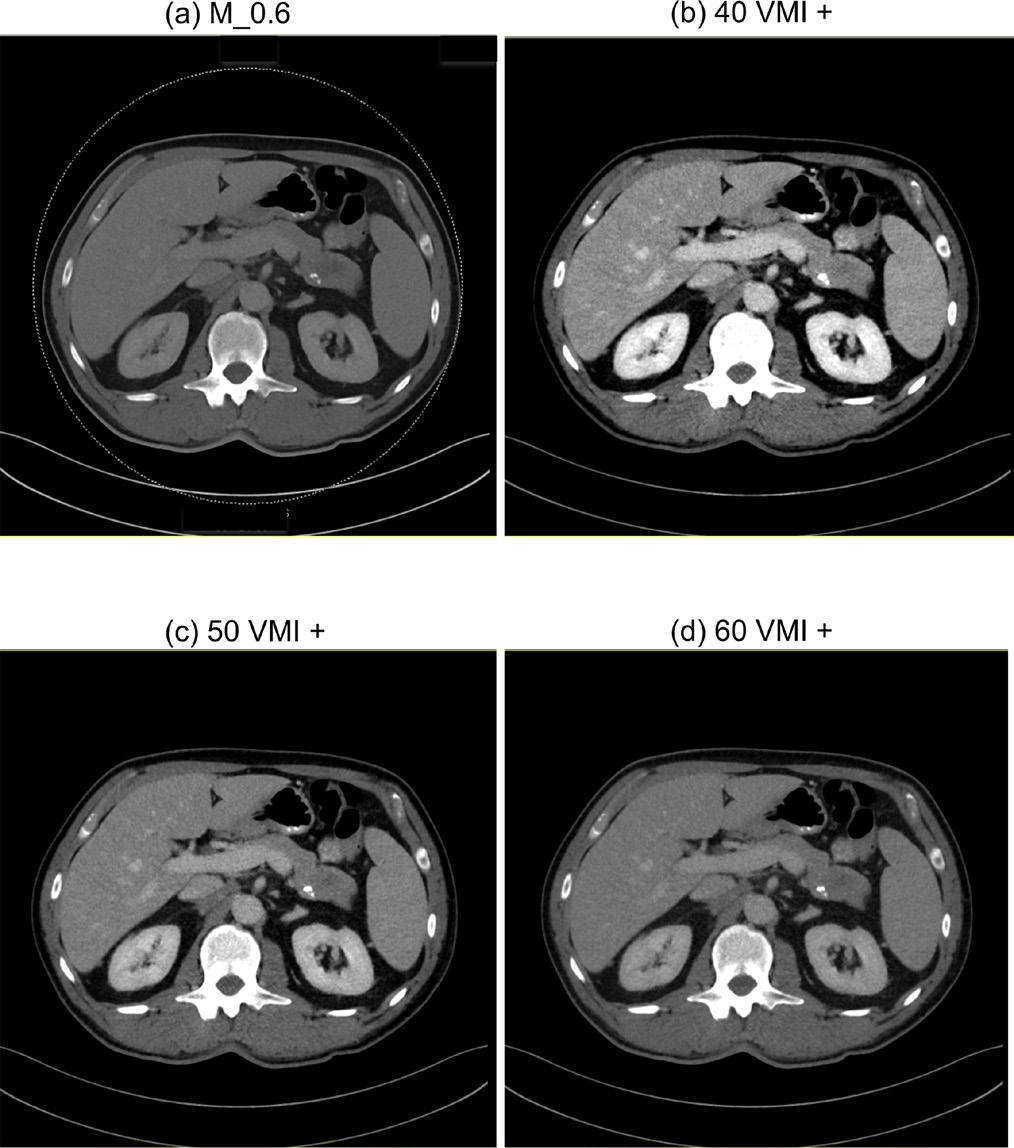
Axial DE-CT images of a 45-year-old male patient. M_0.6 (**a**), 40 VMI+ (**b**), 50 VMI+ (**c**), 60 VMI+ (**d**). Window settings in all reconstructions: level, 56 HU; width 342 HU.

Based on the results of prior studies,^28,29^ we additionally reconstructed VMI+ images at 40 keV, 50 keV and 60 keV on a 3D multimodality workstation (syngo.via, version VB10B, Siemens Healthineers) (Figures 2 and 3). All DECT images were reconstructed in axial orientation with a slice thickness of 3 mm and an increment of 1.5 mm.

### Objective image analysis

An investigator with more than three years of experience in oncological radiology (SM) measured signal attenuation (HU) in intrahepatic veins (preferably left intrahepatic vein), extrahepatic portal vein, liver parenchyma, abdominal aorta, spleen, kidney and psoas muscle. Region of interest (ROI) measurements were drawn as large as possible with a minimum area of 0.5 cm^2^, carefully avoiding surrounding structures. Noise was quantified using the standard deviation (SD) of each measured structure. Signal-to-noise ratio (SNR) was calculated (SNR = HU_region of interest_ / Noise_region of interest_). To analyse image quality objectively, contrast-to-noise ratio (CNR) was calculated using the following formula^14,30^ : CNR = (HU_region of interest_ – HU_muscle_) / Image Noise.

### Subjective image analysis

Three radiologists with more than five years of experience in oncological radiology (JS, SM, LL) assessed image contrast, image noise and overall image quality of abdominal parenchymal and vascular structures including liver parenchyma, spleen, kidney as well as abdominal aorta, intrahepatic veins, and extrahepatic portal vein in standard linearly-blended image series and in the additionally reconstructed VMI+ image series at 40 keV, 50 keV and 60 keV. 5-point Likert scale (1 = very poor, 2 = poor, 3 = fair, 4 = good, 5 = excellent) was used to assess image quality. Ratings ranging from 1 (very poor) to 2 (poor) were defined as non-diagnostic (insufficient), ratings ranging from 3 (fair) to 5 (excellent) were defined as diagnostic (sufficient).

Preset window settings were preset to a width of 800 HU with a level of 300 HU, but could be freely modified by the readers.

All images were analysed in a random order and independently in different readout sessions. All readers were blinded to the reconstruction series. Only one reconstruction series was evaluated during each readout session and an interval of three weeks was kept between analysis of linear blended images and VMI+ images to reduce recall bias.

### Statistical analysis

Statistical analysis was performed using dedicated statistical software (StateCorp. 2019. Stata Statistical Software: Release 16. College Station, TX: StataCorp LLC). The Kolmogorov-Smirnov test was used to analyse data regarding normal distribution. Data showing normal distribution were analysed with t-test. Data showing non-normal distribution were analysed with Wilcoxon Signed-Ranked test.

To calculate interobserver agreement among the three reviewers, Intraclass-Correlation Coefficient (ICC) was used in a two-way mixed-effects model and interpreted as follows: ICC <0.40 = poor agreement, ICC 0.40–0.59 = fair agreement, ICC 0.60–0.79 = substantial agreement, ICC 0.80–1 = excellent agreement.^31^

A *p*-value (*p*) ≤ 0.05 indicated statistical significance.

## Results

### Study population

In total, 262 patients (210 male; mean age 62.6 ± 13.3 years) were included in the study.

Thoracoabdominal DECT radiation metrics in venous phase acquisition were 10.72 ± 4.69 mGy (range, 4.14 mGy – 33.31 mGy) for mean volume CTDI and 745.28 ± 339,09 mGy*cm (range, 268.4 mGy*cm – 2584.8 mGy*cm) for mean DLP.

### Objective image analysis

Attenuation and noise were significantly higher in VMI+ series compared to linearly-blended image series in all evaluated regions with highest values for 40 keV VMI+ series (*p* < 0.001; Table 1).

**Table 1.** Quantitative image analysis

Parameters	A (M_0.6)	B (40 VMI+)	C (50 VMI+)	D (60 VMI+)	*P*-value of pairwise comparison
**Attenuation**					
Liver Parenchyma	92.48 ± 13.09	189.80 ± 44.91	145.28 ± 29.56	117.58 ± 20.68	All,<0.001
Intrahepatic Vein	105.01 ± 15.45	274.67 ± 67.43	198.63 ± 45.18	151.66 ± 30.79	All,<0.001
Spleen	93.60 ± 14.56	215.82 ± 47.95	159.77 ± 31.38	124.14 ± 21.60	All,<0.001
Portal Vein	115.80 ± 21.78	346.92 ± 91.22	240.33 ± 58.82	175.23 ± 38.94	All,<0.001
Kidney	119.81 ± 27.03	379.35 ± 103.03	260.03 ± 67.11	186.65 ± 45.35	All,<0.001
Aorta	119.22 ± 25.65	354.56 ± 96.85	245.20 ± 62.90	178.76 ± 41.97	All,<0.001
Muscle	51.79 ± 7.96	77.13 ± 16.33	65.65 ± 12.00	59.27 ± 9.23	All,<0.001
**Noise**					
Liver Parenchyma	9.10 ± 1.79	22.44 ± 3.80	16.17 ± 2.80	12.48 ± 2.20	All,<0.001
Intrahepatic Vein	10.42 ± 2.20	29.13 ± 6.33	20.98 ± 4.75	15.70 ± 3.49	All,<0.001
Spleen	8.73 ± 1.58	22.40 ± 4.19	16.84 ± 8.99	12.29 ± 2.09	All,<0.001
Portal Vein	10.47 ± 3.69	26.60 ± 5.29	18.99 ± 3.71	14.13 ± 2.68	All,<0.001
Kidney	11.25 ± 2.57	29.17 ± 6.37	21.05 ± 4.53	15.61 ± 3.24	All,<0.001
Aorta	9.52 ± 1.89	26.53 ± 6.26	19.10 ± 3.73	14.24 ± 2.51	All,<0.001
Muscle	9.14 ± 1.62	22.49 ± 4.41	16.44 ± 3.16	12.77 ± 2.39	All,<0.001
**SNR**					
Liver Parenchyma	10.59 ± 2.84	8.69 ± 2.56	9.24 ± 2.51	9.68 ± 2.28	All,<0.001
Intrahepatic Vein	10.49 ± 2.59	9.64 ± 2.50	9.73 ± 2.47	9.98 ± 2.53	A *vs* B,<0.001; A *vs* C,<0.001 A *vs* D, 0.0017; B *vs* C, 0.3976 B *vs* D, 0.0076; C *vs* D, 0.0165
Spleen	11.11 ± 2.76	9.92 ± 2.75	10.04 ± 2.54	10.36 ± 2.37	B *vs* C, 0.1974; All other,<0.001
Portal Vein	11.65 ± 3.21	13.34 ± 3.97	12.89 ± 3.22	12.70 ± 3.25	B *vs* C, 0.089; B *vs* D, 0.0011 C *vs* D, 0.1729; All other,<0.001
Kidney	11.07 ± 3.28	13.33 ± 3.94	12.68 ± 3.61	12.32 ± 3.60	C *vs* D, 0.0043; All other,<0.001
Aorta	12.99 ± 3.82	13.70 ± 3.72	13.16 ± 3.62	12.85 ± 3.45	A *vs* B, 0.0021; A *vs* C, 0.4330 A *vs* D, 0.4998; C *vs* D, 0.0088 All other,<0.001
Muscle	60.93 ± 7.83	99.62 ± 17.23	82.09 ± 12.34	72.04 ± 9.10	All,<0.001
**CNR**					
Liver Parenchyma	4.99 ± 2.11	6.44 ± 2.97	6.13 ± 2.68	5.73 ± 2.39	B *vs* C 0.0019; All other,<0.001
Intrahepatic Vein	6.52 ± 2.50	11.26 ± 4.71	10.23 ± 4.04	9.06 ± 3.45	All,<0.001
Spleen	5.11 ± 2.28	8.02 ± 3.52	7.30 ± 2.90	6.37 ± 2.48	All,<0.001
Portal Vein	7.84 ± 3.26	15.50 ± 6.70	13.49 ± 5.37	11.41 ± 4.48	All,<0.001
Kidney	8.35 ± 3.86	17.37 ± 7.48	15.07 ± 6.10	12.59 ± 5.16	All,<0.001
Aorta	8.28 ± 3.84	16.03 ± 7.30	14.00 ± 6.08	11.86 ± 5.06	All,<0.001

*Results of attenuation, noise, signal-to-noise ratio (SNR) and contrast-to-noise ratio (CNR) including mean scores ± standard deviation across areas of interest (liver parenchyma, intrahepatic vein, spleen, portal vein, kidney, aorta, muscle).*

Highest SNR for portal vein, kidney, aorta and muscle were measured in 40 keV VMI+ image series (portal vein, 13.3 ± 4.0; kidney, 13.3 ± 3.9; aorta, 13.7 ± 3.7; muscle, 99.6 ± 17.2) with significantly higher values compared to the linearly-blended image series (portal vein, 11.7 ± 3.2, kidney 11.1 ± 3.2, aorta 13.0 ± 3.8, muscle 60.9 ± 7.8; *p* < 0.001).

For liver parenchyma, intrahepatic veins and spleen, linearly-blended image series showed higher SNR (liver parenchyma, 10.6 ± 2.8, intrahepatic vein 10.5 ± 2.6, spleen 11.1 ± 2.8) compared to the reconstructed VMI+ series (*p* < 0.001)

Detailed quantitative measurements for attenuation, noise and SNR values are displayed in Table 1.

In all evaluated anatomical areas, CNR values were significantly higher in VMI+ image series compared to linearly-blended image series (Table 1; *p* < 0.001). Of those, CNR values across all areas of interest were significantly higher in 40 keV VMI+ image series (*e.g.,* liver parenchyma, 6.4 ± 3.0) compared to 50 keV (liver parenchyma, 6.1 ± 2.7, *p* = 0.014; all other *p* < 0.001) and 60 keV VMI+ image series (liver parenchyma 5.7 ± 2.4, *p* < 0.001).

**Figure 4.**
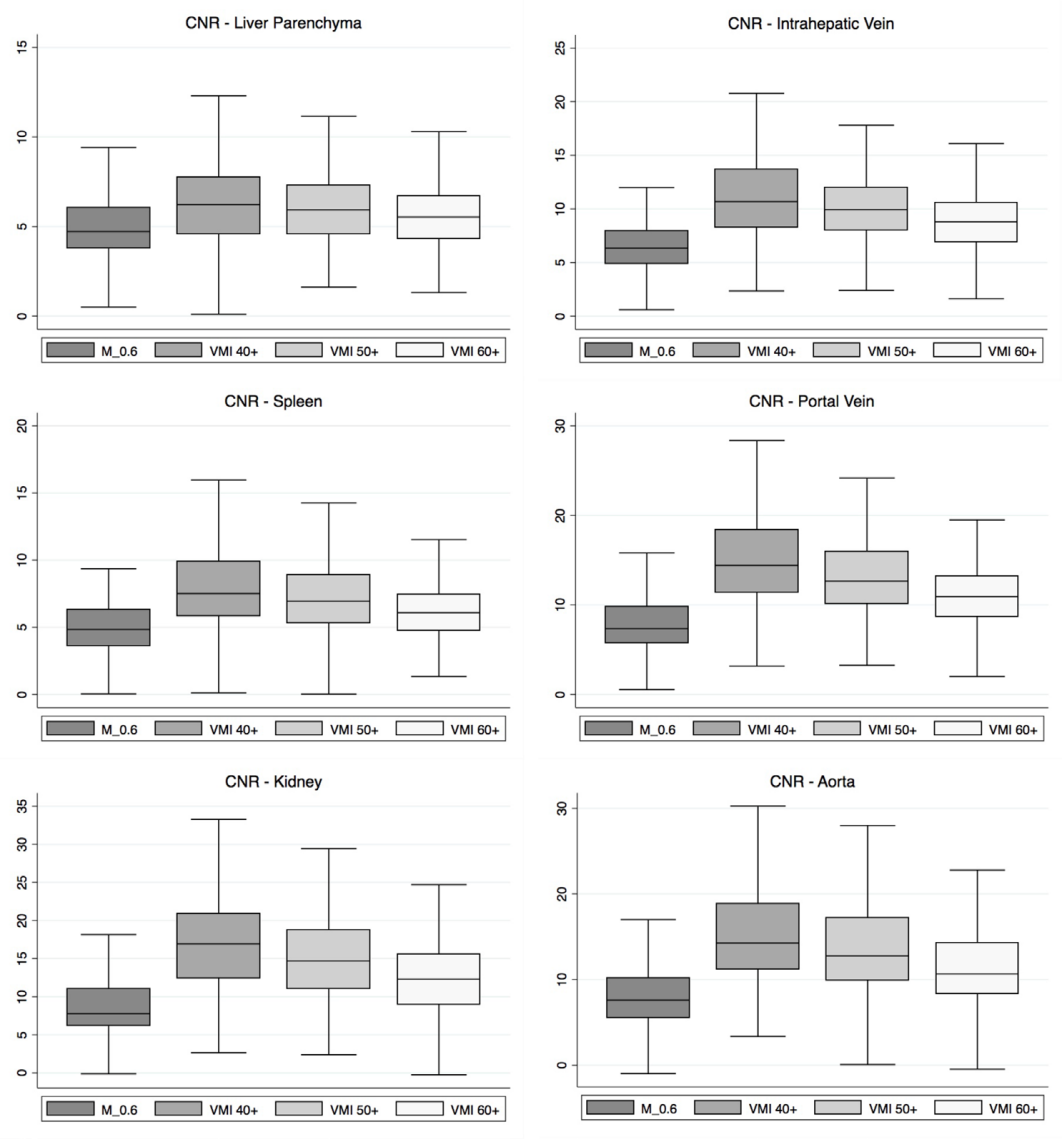
Box-and-whisker plots of contrast-to-noise ratio across areas of interest

Detailed quantitative measurements for CNR are displayed in Table 1. Box-Whisker-Plots for CNR values are provided in Figure 4.

### Subjective image analysis

Best overall image quality was seen in 50 VMI+ image series with significantly higher rating values compared to all other assessed image series (median, 4; interquartile range (IQR), 4–5; *p* < 0.001). For overall image quality in the 50+ VMI reconstructions, excellent interobserver agreement (ICC, 0.82) was achieved.

Subjective ratings for image contrast were significantly higher in all VMI+ image series compared to linearly-blended image series with 40 VMI+ rated highest (median, 5; IQR, 5–5; *p* < 0.001) with a substantial interobserver agreement (ICC, 0.74).

For image noise, 60 keV VMI+ images were rated highest (median, 4; IQR, 4–5) from all VMI+ image series with significantly better ratings compared to 40 keV (median, 3; IQR, 3–3) and 50 keV (median, 4; IQR, 3–4) VMI+ images (*p* < 0.001).

While 36.9% of the studies were rated as non-diagnostic (insufficient) in terms of image contrast when using linearly-blended image reconstruction, only 1.2% of all studies were rated non-diagnostic (insufficient) when using 50 keV VMI+ image reconstruction.

**Figure 5.**
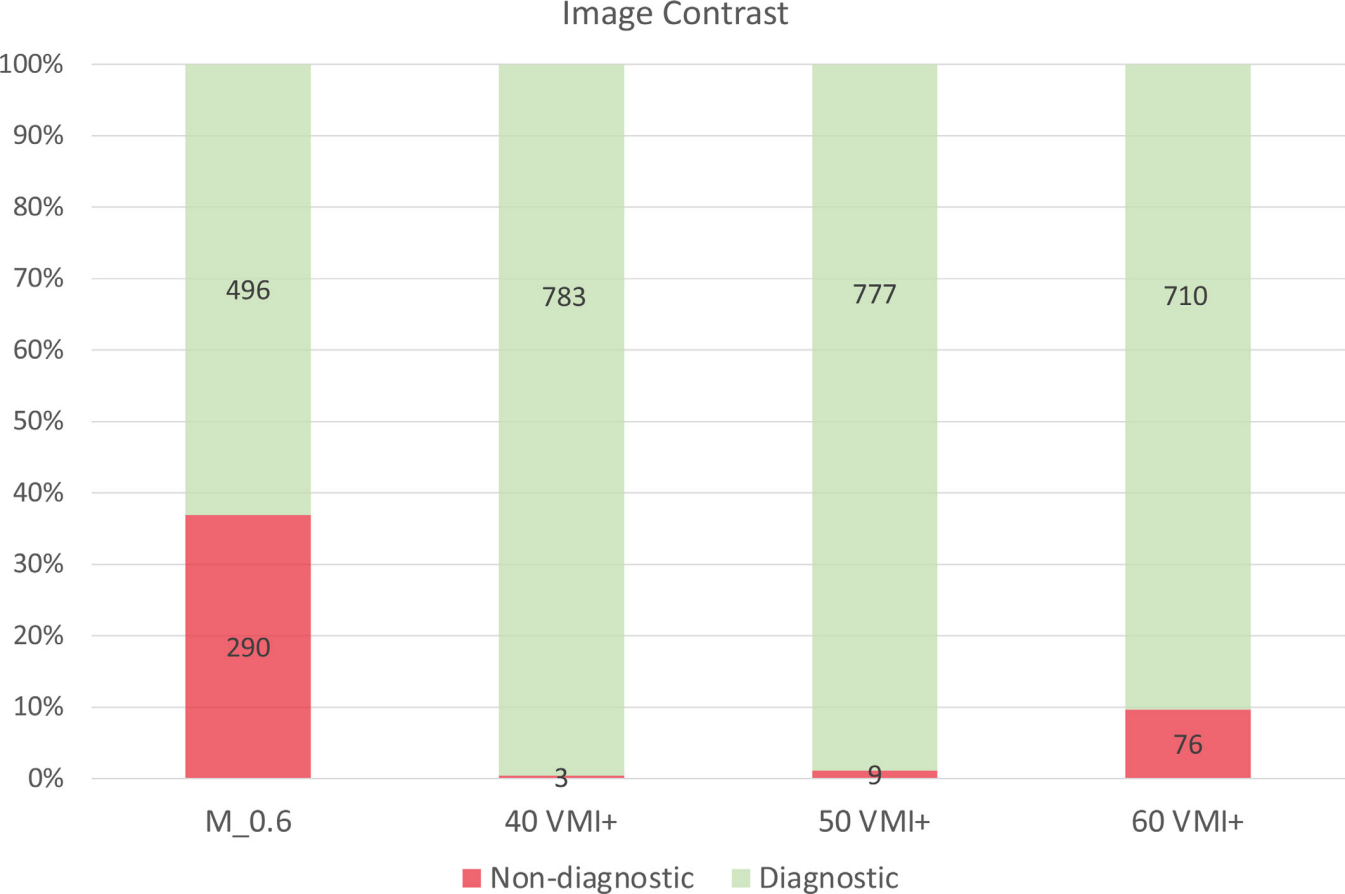
Absolute and relative numbers of subjective ratings for linearly-blended image series and VMI+ images based on the results of qualitative image analysis using 5-point Likert scale. Ratings ranging from 1 (very poor) to 2 (poor) were defined as non-diagnostic (insufficient), ratings ranging from 3 (fair) to 5 (excellent) were defined as diagnostic (sufficient). The Y-axis demonstrates the absolute numbers and relative proportion of diagnostic (sufficient) and non-diagnostic (insufficient) scans in terms of image contrast.

Absolute and relative numbers of subjective ratings for linearly-blended image series and VMI+ images regarding diagnostic (sufficient) and non-diagnostic (insufficient) image contrast are displayed in detail in Figure 5.

## Discussion

The aim of this retrospective study was to evaluate the impact of additional VMI+ image reconstruction in abdominal DECT exams of originally standard linearly-blended CT exams with impaired, non-diagnostic portal-venous contrast in a large cohort of a major tertiary care hospital.

The results of this study are in line with prior smaller studies that have shown that VMI+ image reconstruction improved objective and subjective image quality compared to standard-linearly-blended image series. Its usage can salvage poorly attenuated DECT studies, enabling diagnostic contrast and reducing number of non-diagnostic examinations.

While VMI+ images at 40 keV showed highest CNR values, subjective evaluation favoured 50 keV VMI+ image series with best overall image quality and excellent balance between image noise and contrast.

**Table 2.** Qualitative image analysis

Parameters	A (M_0.6)	B (40 VMI+)	C (50 VMI+)	D (60 VMI+)	*P*-value
**Image Contrast**	3 (2–4)	5 (5–5)	5 (4–5)	4 (3–4)	All,<0.001
*ICC for Image Contrast*	0.91 (0.88–0.93)	0.74 (0.66–0.80)	0.84 (0.80–0.88)	0.89 (0.84–0.92)	
**Image Noise**	5 (4–5)	3 (2–3)	4 (3–4)	4 (4–5)	All,<0.001
*ICC for Image Noise*	0.63 (0.49–0.73)	0.76 (0.67–0.82)	0.82 (0.75–0.87)	0.71 (0.64–0.76)	
**Overall Image quality**	3 (3–4)	3 (3–4)	4 (4–5)	4 (3–4)	A *vs* B, 0,3966 All other,<0.001
*ICC for Overall Image Quality*	0.85 (0.81–0.88)	0.77 (0.72–0.82)	0.82 (0.73–0.87)	0.81 (0.77–0.85)	
*Results of qualitative analysis including median of image contrast, image noise and overall image quality for M_0.6 images, 40 VMI+, 50 VMI+ and 60 VMI+ images. Corresponding interquartile ranges (IQR) are given in brackets [VMI, virtual monoenergetic images; VMI+, noise-optimised VMI]*.					

Prognoses and treatment options of malignant tumours often depend on vascular infiltration. Therefore, high-resolution and artifact-free imaging of vascular structures is of high clinical relevance in oncological imaging.^32,33^ De Cecco et al demonstrated that the application of VMI+ can improve lesion delineation and characterisation as an approach to affect evaluation of response to cancer treatment.^34^

In smaller cohorts, impact of VMI+ on imaging of upper abdominal organs has been evaluated showing that VMI+ reconstruction at an energy level of 50 keV improved detection of hypervascular liver lesions.^19^ Similar findings were presented for the detection of hypoattenuating liver lesions by Caruso et al, who suggested an optimal energy level of 40 keV.^35^ The same energy level was suggested for lesion delineation in renal tumours^17^ and for the detection of peritoneal metastasis.^36^ Beside oncological imaging, several studies have shown that improved image contrast translates into superior diagnostic performance in vascular DECT studies.^29,37,38^ In accordance to the findings of upper abdominal organs, several studies with smaller study cohorts have demonstrated that usage of low keV VMI+ series improves contrast of abdominal vessels including mesenterial arteries and intrahepatic veins.^14,28^

Based on the findings of these smaller scaled studies, we retrospectively reconstructed VMI+ images at low keV energy levels ranging from 40 keV to 60 keV. Our results in objective analysis are consistent with prior evidence taking a large cohort into account and confirming highest CNR values in 40 keV VMI+ image series.

Subjective analysis supported the improved image contrast in the low keV energy levels with highest contrast in the 40 keV VMI+ image series. High contrast properties were also determined in the 50 keV VMI+ image series while having higher overall image quality due to lower image noise levels. As the 50 keV VMI+ series performed best in subjective analysis maintaining high diagnostic contrast, we support the additional low keV VMI+ image reconstruction – particularly 50 keV – in oncologic CT-studies of the abdomen to enhance scan efficacy.

Our data proves that reconstruction of 50 keV VMI+ images significantly reduces the number of non-diagnostic scans in terms of image contrast. In total, 96,9% of non-diagnostic scans acquired with standard linearly-blended imaging could be salvaged by reconstructing 50 keV VMI+ images. Since there is a limitation in administration of contrast agency doses, especially obese patients and patients with kidney failure may benefit from improved image contrast in poorly attenuated studies, possibly avoiding repeatedly performed scans. Also, improved contrast properties using VMI+ reconstruction could facilitate reduction of iodine-based contrast media, which is associated with acute kidney injury in patients with renal insufficiency.^9^

The study has several limitations, which have to be taken into account.

First, the contrast media protocol for cancer staging purposes in our institute with a minimum flow of 2 ml s^−1^ is variable. An exact comparison of all poorly opacified studies is limited in cases where higher flow rates of contrast media were injected as the injection rate affects timing of contrast enhancement.^1,39^

Second, our institute works with the dual-source DECT system. As a consequence, our technical approach for VMI+ may restrict the application of our results to users of dual-source DECT systems. Further studies are necessary to confirm our results for rapid kV-switching or dual-layer DECT systems.^40^

Last, we used the same abdominal DECT scan for baseline image series and for reconstruction of VMI+ image series and recall bias may have occurred.

## Conclusions

In conclusion, this study demonstrates that VMI+ images can salvage the majority of poorly opacified abdominal portal-venous CT exams. We recommend reconstruction of 50 keV VMI+ image series to increase image contrast and image quality in poorly enhanced CT studies of the abdomen for cancer staging purposes obtained in clinical routine.
